# MiR-9, miR-153 and miR-124 are down-regulated by acute exposure to cocaine in a dopaminergic cell model and may contribute to cocaine dependence

**DOI:** 10.1038/s41398-018-0224-5

**Published:** 2018-08-30

**Authors:** Judit Cabana-Domínguez, Concepció Arenas, Bru Cormand, Noèlia Fernàndez-Castillo

**Affiliations:** 1 Departament de Genètica, Microbiologia i Estadística, Facultat de Biologia, Universitat de Barcelona, Barcelona, Catalonia Spain; 2 Centro de Investigación Biomédica en Red de Enfermedades Raras (CIBERER), Instituto de Salud Carlos III, Madrid, Spain; 3 Institut de Biomedicina de la Universitat de Barcelona (IBUB), Barcelona, Catalonia Spain; 4 Institut de Recerca Sant Joan de Déu (IR-SJD), Esplugues de Llobregat, Catalonia Spain

## Abstract

Cocaine is one of the most used psychostimulant drugs worldwide. MicroRNAs are post-transcriptional regulators of gene expression that are highly expressed in brain, and several studies have shown that cocaine can alter their expression. In a previous study, we identified several protein-coding genes that are differentially expressed in a dopaminergic neuron-like model after an acute exposure to cocaine. Now, we used the prediction tool WebGestalt to identify miRNA molecules potentially involved in the regulation of these genes. Using the same cellular model, we found that seven of these miRNAs are down-regulated by cocaine: miR-124-3p, miR-124-5p, miR-137, miR-101-3p, miR-9-5p, miR-369-3p and miR-153-3p, the last three not previously related to cocaine. Furthermore, we found that three of the miRNA genes that are differentially expressed in our model (hsa-miR-9-1, hsa-miR-153-1 and hsa-miR-124-3) are nominally associated with cocaine dependence in a case–control study (2,085 cases and 4,293 controls). In summary, we highlighted novel miRNAs that may be involved in those cocaine-induced changes of gene expression that underlie addiction. Moreover, we identified genetic variants that contribute to cocaine dependence in three of these miRNA genes, supporting the idea that genes differentially expressed under cocaine may play an important role in the susceptibility to cocaine dependence.

## Introduction

Cocaine is one of the most used illicit drugs and its use is one of the major public health problems worldwide^1^. However, only a subset of individuals exposed to cocaine (around 15–16% of cocaine users) develop an addiction^2^.

Transcriptomic studies performed in animal models and human brain post-mortem samples have revealed that both acute and chronic exposure to cocaine produce changes in the expression of genes related to diverse functional categories, such as cell adhesion, extracellular matrix, synaptic communication and neuroplasticity, receptors, ion channels and transporters, oligodendrocytes and myelin, mitochondrial function, apoptosis and cell death, transcription factors and signal transduction^3,4^. Cocaine also produces epigenetic adaptations like changes in DNA methylation, chromatin remodeling or alterations in miRNA regulation^5–7^. Both epigenetic adaptations and changes in gene expression induced by cocaine trigger molecular and cellular adaptations in the central nervous system that may explain the persistence of drug-seeking behavior, even after extended periods of abstinence^4,8–10^.

The interaction between the individual’s genetic background, epigenetic factors and environment^11^ determine how neuronal circuits adapt to chronic cocaine exposure, establishing the development of addiction in some individuals but not in others^10^. Cocaine dependence is one of the most heritable psychiatric disorders (around 65–79%^12–15^), and some of those genetic risk factors may lie in genes that mediate cocaine’s effects, conferring initial vulnerability to the establishment of drug-induced neuroadaptations.

MicroRNAs (miRNAs) are post-transcriptional regulators of gene expression that bind to target mRNAs to inhibit translation or promote mRNA degradation. Each miRNA can regulate the expression of hundreds of different mRNAs, and each mRNA species can be targeted by several miRNAs. MiRNA regulation generates a very complex and dynamic system that allows the cells to fine-tune gene expression^16–18^. In addition to the canonical cytoplasmic function, there is evidence that miRNAs located in the nucleus can regulate mRNA stability in the nucleolus and modulate alternative splicing, as well as activate or inhibit the transcription of target genes^19^.

MiRNAs are very abundant in the central nervous system, especially in the synapto-dendritic compartment, where they control local mRNA translation in response to neuronal activity^20^, which is essential for synapses development, neuronal plasticity, memory and learning^21,22^. It is known that cocaine alters miRNA expression profiles in brain^23^. Using RNAseq, Eipper-Mains and collaborators inspected cocaine-responsive miRNAs in nucleus accumbens (NAc) and striatal post-synaptic densities (PSDs) in chronically cocaine-treated mice. They found several differentially expressed miRNAs, most of them belonging to four miRNA families (miR-8, miR-7, miR-142 and Let-7), which suggests that cocaine modulates expression of miRNAs that have similar target genes^24^. In a subsequent study, they used a list of mRNAs^25^ found enriched in PSD to identify predicted targets of these miRNAs^26^, using bioinformatic tools.

In a previous study we evaluated changes in gene expression induced by acute cocaine exposure in a dopaminergic neuron-like model (differentiated SH-SY5Y cells). We found differences in 756 protein-coding genes involved in several processes with potential relevance to central nervous system function, including regulation of transcription, chromatin modification, focal adhesion and cell projection, and neurotrophin and MAPK signaling pathways^27^. Furthermore, these genes showed an enrichment of predicted binding sites for several miRNAs, miR-124 among them. Based on these results, we hypothesized that the observed changes in gene expression can be produced, in part, by changes in miRNA expression. Here, we aimed at validating predicted changes in miRNA expression altered by cocaine in differentiated SH-SY5Y cells. Furthermore, we aimed to evaluate their possible contribution to the susceptibility to cocaine dependence through a case–control association study with common genetic variants.

## Materials and methods

### MiRNA selection

In a previous study^27^ we assessed transcriptomic changes induced by cocaine in a human dopaminergic neuron-like model (differentiated SH-SY5Y neuroblastoma cells) at 6 and 24 h after an acute 30-min exposure to cocaine at 1 or 5 μM. We identified 756 protein-coding genes showing differential expression only under 5 μM cocaine after 6 h (10% FDR), 337 of them down-regulated and 419 up-regulated^27^. In the present study we further evaluated the enrichment of miRNA-binding sites among the differentially expressed genes. We performed a “miRNAs target analysis” with the online tool WEB-based GEne SeT AnaLysis Toolkit (WebGestalt, http://www.webgestalt.org/webgestalt_2013)^28,29^ using the default settings and considering up-regulated and down-regulated subsets of genes separately (Supplementary Table 1). We filtered the results obtained using the following criteria: (i) miRNAs expressed in brain, according to the miRIAD database (http://bmi.ana.med.uni-muenchen.de/miriad/), (ii) every single miRNA has predicted binding sites in at least 10 target genes, (iii) an enrichment *P-*value <0.01. We also discarded predictions of miRNA families due to the difficulty to validate all the different miRNAs within them. We then prioritized miRNAs which predicted target genes were enriched in any biological function according to GO or KEGG, or miRNAs with a reported relation with cocaine.

### MiRNA expression

Changes in the expression of miRNA genes were tested in new cultures of dopaminergic neuron-like cells exposed to cocaine. SH-SY5Y cells (ATCC, LGC Standards) were differentiated and validated as previously described^27^. After an exposure to 5 µM cocaine for 30 min, the medium was replaced and cells were retrieved at 3 or 6 h, performing 10 biological replicates per condition. Total RNA including miRNAs was isolated using the miRNeasy Micro Kit (Qiagen, Hilden, Germany). RNA concentration was determined using the NanoDrop ND-1000 spectrophotometer (NanoDrop Technologies, TermoFisher Scientific Inc., Wilmington, DE, USA) and integrity was evaluated with the Bioanalyzer 2100 platform (Agilent Technologies, Santa Clara, CA, USA). The averaged RIN values (RNA Integrity Number) was 9.98, being 9.8 the lowest one.

Mature miRNAs were retrotranscribed using the miScript II RT Kit (Qiagen) and real-time quantitative PCR (qRT-PCR) experiments were performed with the miScript SYBR Green PCR Kit (Qiagen) in the LightCycler 480 II system (Roche Life Sciences, Branford, CT, USA). For primers sequences, see Supplementary Table 2. To select a reference gene we tested several miRNA genes, and compared and ranked them using the online tool RefFinder (http://150.216.56.64/referencegene.php?type=reference) that integrates different computational tools (geNorm, Normfinder, BestKeeper and the comparative ΔCt method). Relative quantification was performed for each miRNA by normalizing the level of expression to that of the selected reference miR-3911, which is stable across samples.

A one-way ANOVA with Dunnett’s post hoc test was performed to examine the gene expression changes among groups. Previously, normality and homogeneity of variance were ascertained in our samples using the Shapiro–Wilk and Bartlett tests, respectively. A value of *P* < 0.05 was considered to be statistically significant.

### Gene networks

The differentially expressed miRNAs were subjected to analysis of gene networks using the Ingenuity Pathway Analysis 8.8 software (http://www.ingenuity.com/products/ipa; Ingenuity Systems, Redwood city, CA, USA).

### Gene-based association analysis

We used MAGMA 1.05b^30^ to perform a gene-based association analysis to test the contribution to cocaine dependence susceptibility of the miRNAs which expression is altered by cocaine. We used the summary statistics from a GWAS meta-analysis of cocaine dependence (*N*_cases_ = 2,085; *N*_controls_ = 4,293)^31^, considering the SNP-wise mean model, in which the sum of −log(SNP *P-*value) for SNPs located within a gene and its regulatory regions was used as the statistic test. We selected all miRNA genes encoding each differentially expressed mature miRNA and the regulatory regions, including both that of the miRNA and that of the host protein-coding gene, identified according to histone marks H3K4me1, H3K4me3, H3K27me3 and H3K27Ac in UCSC Genome Browser (assembly GRCh37/hg19)^32^. The gene *P-*value was calculated using a known approximation of the sampling distribution^33^. MAGMA accounts for gene size, number of SNPs in a gene and linkage disequilibrium between markers (estimated using data from 1000Genomes Phase 3, European ancestry samples^34^).

Summary statistics used in this analysis derive from a case–control GWAS meta-analysis of cocaine dependence performed using four datasets from the dbGaP repository (https://www.ncbi.nlm.nih.gov/gap) under the project 10608. All cases used met DSM-IV criteria for cocaine dependence, although most of them are also dependent to other drugs of abuse. Control individuals were taken from the general population, except for those of the SAGE sample, where drug abuse or dependence were formally discarded^31^.

## Results

In a previous study we identified 756 protein-coding genes showing differential expression in a dopaminergic neuron-like model after an acute exposure to cocaine^27^. Here we evaluated whether these changes in gene expression might be produced by changes in miRNA expression induced by cocaine.

Using the online prediction tool WebGestalt we tested enrichment for miRNA-binding sites in the differentially expressed mRNAs. We found enrichment for 6 miRNAs in the subset of down-regulated target genes, and 84 miRNAs for the up-regulated ones (Supplementary Table 1). After filtering them according to the criteria described above (see Materials and methods), we selected 11 miRNAs (hsa-miR-9-3p, hsa-miR-9-5p, hsa-miR-101-3p, hsa-miR-105-5p, hsa-miR-124-3p, hsa-miR-124-5p, hsa-miR-137, hsa-miR-153-3p, hsa-miR-181-5p, hsa-miR-186-5p and hsa-miR-369-3p), all of them predicted to bind up-regulated genes in our previous study. Thus, we expected to find these miRNAs down-regulated, as they are negative regulators of gene expression.

**Fig. 1:**
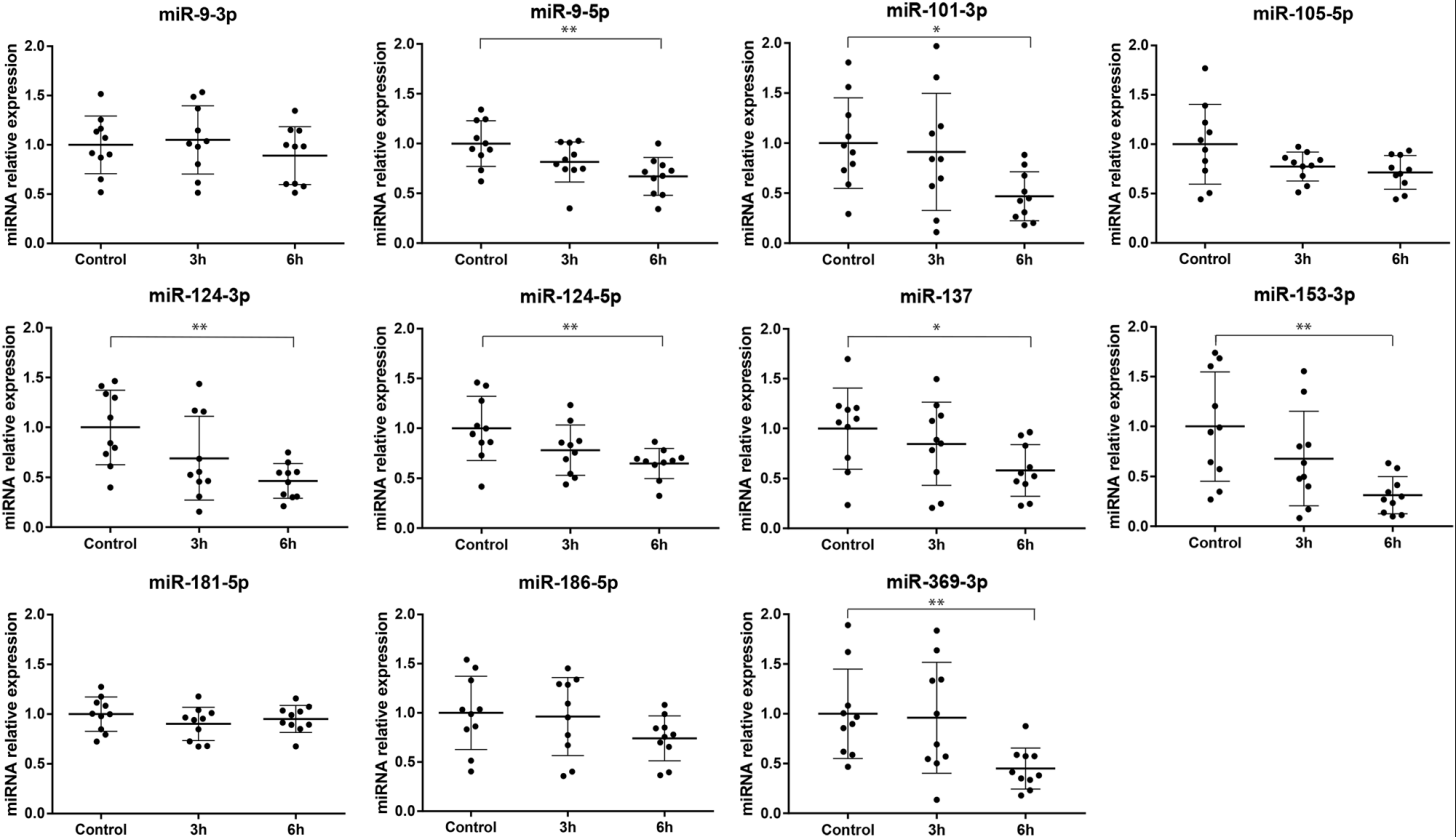
Transcription levels of all tested miRNAs determined by qRT-PCR at 3 and 6 h after a 30-min exposure to 5 µM cocaine in dopaminergic neuron-like cells. Significant differences compared to control cells (not exposed to cocaine) normalized to miR-3911 are indicated (*P-value <0.05, **P-value <0.01). Mean and standard deviation are shown

Since the RNA samples used in the previous work did not contain miRNAs, we performed new experiments of cocaine exposure in differentiated SH-SY5Y cells, to experimentally validate changes in miRNA expression, at 3 and 6 h after 30 min of cocaine treatment at 5 µM. The results showed that 6 h after the 30-min cocaine treatment, seven out of the 11 tested miRNAs were down-regulated, as predicted, when compared to untreated control cells: miR-9-5p (FC = −1.49, SE = 0.19, *P* = 1.3e-03), miR-101-3p (FC = −2.00, SE = 0.26, *P* = 0.012), miR-124-3p (FC = −2.17, SE = 0.17, *P* = 1.4e-03), miR-124-5p (FC = −1.54, SE = 0.15, *P* = 4e-03), miR-137 (FC = −1.69, SE = 0.26, *P* = 0.015), miR-153-3p (FC = −2.94, SE = 0.2, *P* = 1.3e-03) and miR-369-3p (FC = −2.22, SE = 0.2, *P* = 7.6e-03) (Fig. 1). None of them showed significant changes at 3 h after treatment, although in some cases a decreasing trend was observed (Fig. 1). The other four miRNAs did not change their expression significantly (Fig. 1).

**Fig. 2:**
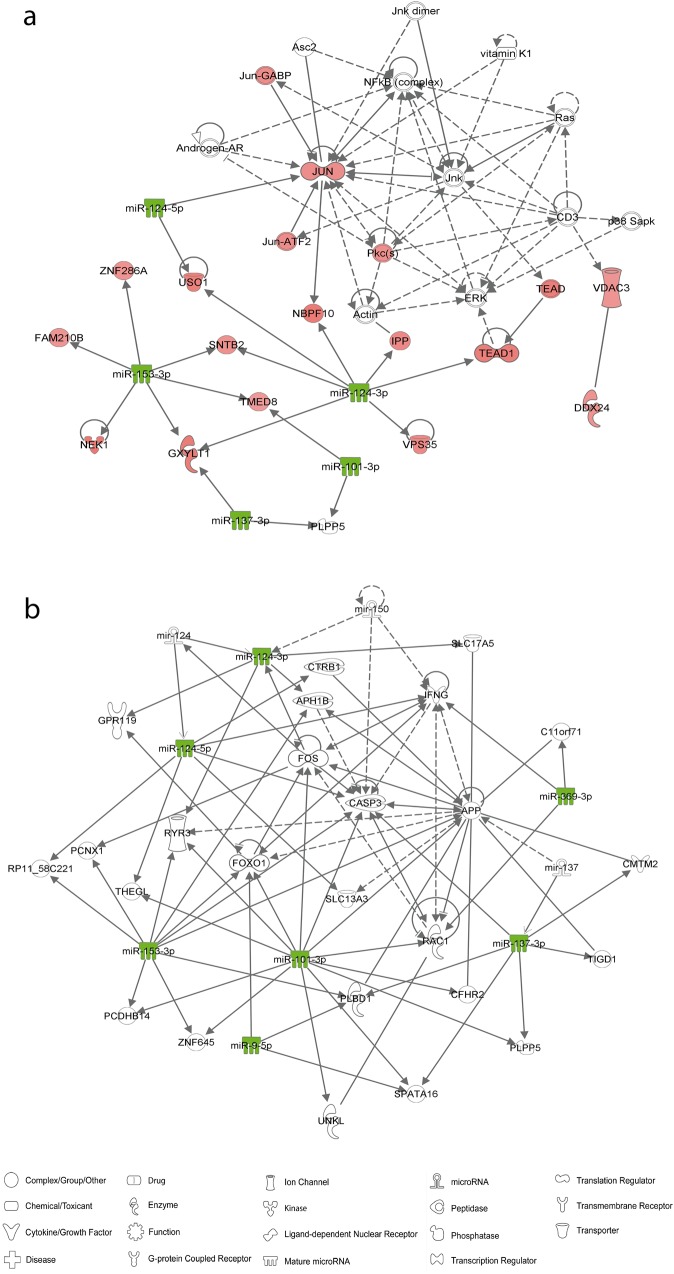
Gene networks involved in **a)** 'developmental disorder, ophthalmic disease, organismal injury and abnormalities' (score = 41) and **b)** 'cell-to-cell signaling and interaction, nervous system development and function, inflammatory disease' (score = 21). The green and red nodes in the pathway indicate the down- and up-regulated genes, respectively

Then, we evaluated the relationship between these miRNAs and other genes by gene network analysis. First, we inspected the connections between the identified down-regulated miRNAs and their up-regulated target mRNA (predicted to contain miRNA-binding sites). We found five out of the seven differentially expressed miRNAs connected with 17 up-regulated genes in a network related to “Developmental Disorder, Ophthalmic Disease, Organismal Injury and Abnormalities” (score = 41; Fig. 2a). Second, we inspected how these differentially expressed miRNAs are connected with other genes in the genome and found that all seven miRNAs were present and highly connected in a network involved in “Cell-To-Cell Signaling and Interaction, Nervous System Development and Function, Inflammatory Disease” (score = 21; Fig. 2b), including target genes such as *FOS*, *CASP3* and *RYR3*.

Finally, we evaluated the possible contribution of these miRNAs to cocaine dependence susceptibility by assessing the presence of common genetic risk variants for cocaine dependence within the genes encoding these miRNAs. Thus, we performed a gene-based association analysis in a sample of 2,085 cocaine-dependent individuals and 4,293 controls. Interestingly, three miRNA genes were found nominally associated with cocaine dependence: hsa-miR-9-1 (*P* = 0.011), hsa-miR-153-1 (*P* = 0.036) and hsa-miR-124-3 (*P* = 0.042) (Table 1). These genes encode mature miRNAs that are highly connected in the identified networks (Fig. 2a, b).

**Table 1:** Results of miRNA gene-based association analysis of common genetic variants in a GWAS meta-analysis of cocaine dependence in individuals of European ancestry

**Gene ID**	**Coordinates** ^**a**^	**N SNPs**	**ZSTAT**	***P-*** **value**	**SNP ID**	**Risk allele**	**SNP** ***P-*** **value**
hsa-miR-101-1	chr1:65523406-65534997	19	0.2260	0.4105	rs486378	G	0.0420
hsa-miR-101-2	chr9:4828718-4857394	146	0.4890	0.3123	rs7870037	A	0.0113
hsa-miR-124-1	chr8:9760113-9764675	6	−0.8845	0.8117	rs62489494	C	0.7111
hsa-miR-124-2	chr8:65283014-65291854	17	0.3783	0.3525	rs190938	G	0.0518
**hsa-miR-124-3**	**chr20:61804035-61811005**	**18**	**1.7294**	**0.0418**	**rs76137972**	**G**	**0.0342**
hsa-miR-137	chr1:98509277-98521215	30	−0.1075	0.5428	rs12744323	C	0.1661
**hsa-miR-153-1**	**chr2:220158748-220161492**	**8**	**1.7939**	**0.0364**	**rs6436132**	**T**	**0.0178**
hsa-miR-153-2	chr7:157362028-157377114	38	−0.7436	0.7714	rs221296	A	0.2219
hsa-miR-153-2_HG^b^	chr7:158377426-158412766	123	0.6251	0.2659	rs12670306	A	0.0221
hsa-miR-369	chr14:101522238-101541542	78	0.8836	0.1884	rs12431682	T	5.3E-03
**hsa-miR-9-1**	**chr1:156389579-156394148**	**5**	**2.2778**	**0.0113**	**rs78605853**	**G**	**6.0E-03**
hsa-miR-9-2	chr5:87957415-87991755	57	0.6955	0.2433	rs201864123	G	0.0484
hsa-miR-9-3	chr15:89899916-89911697	27	−0.6557	0.7440	rs961288	C	0.4393

Analysis performed using 2,085 cases and 4,293 controls

^a^Gene coordinates based on GRCh37/hg19. NSNPs: number of SNPs per region included in the gene-based analysis; ZSTAT: the *Z*-value for the gene; SNP ID: SNP with lowest *P*-value

^b^Promoter region of miR-153-2 host gene (*PTPRN2*). In bold, nominally associated genes

## Discussion

In this study we explored the possibility that cocaine-induced changes in gene expression found in a previous study of our group may be triggered by changes in the expression of miRNA molecules.

We used prediction tools to identify miRNAs that potentially regulate protein-coding genes which expression is altered by cocaine according to a previous study by our group in a dopaminergic cell model^27^. Seven of the 11 miRNAs selected were significantly down-regulated after an acute exposure to cocaine (miR-9-5p, miR-101-3p, miR-124-3p, miR-124-5p, miR-137, miR-153-3p and miR-369-3p), consistent with the predictions and with the up-regulation of their target genes. Moreover, through gene network analyses, we correlated down-regulated miRNAs with the up-regulated target genes identified in our previous study. This network includes several genes previously associated with cocaine dependence, such as *PKC*, encoding an enzyme involved in cocaine-induced neuroplasticity^35–37^, or *JUN*, an early immediate gene (EIG) that activates transcription through heterodimerization at AP-1 sites^38–41^. Furthermore, we found interaction between miR-124-3p and its validated target *TEAD1*^42,43^, encoding a transcription factor regulated by the Hippo pathway that controls proliferation^44^. On the other hand, we performed a network analysis to test the interaction of the seven down-regulated miRNAs identified with all the genes in the genome. We identified an interesting network involved in “Cell-To-Cell Signaling and Interaction, Nervous System Development and Function, Inflammatory Disease” in which the seven differentially expressed miRNAs are present and highly interconnected. In this network we highlight three genes: *FOS, CASP3* and *RYR3*. *FOS* is a very important EIG highly associated with cocaine dependence, as recently reviewed elsewhere^45^. *CASP3* encodes a pro-apoptotic protein involved in cocaine-mediated cell death that is very important for both adult and fetal cocaine neurotoxicity^46,47^. And *RYR3*, a member of the RyR family involved in cocaine-induced place preference regulated by dopamine D1 receptor^48.^ *RYR3* is a highly confident predicted target of miR-124-3p and miR-153-3p according to different online miRNA:mRNA prediction tools like miRBD, DianaT, TargetScan, and PicTar. Furthermore, *RYR3* was found up-regulated in dopaminergic-enriched regions from substantia nigra and ventral tegmental area in post-mortem brain samples from cocaine abusers^38^, as well as in our previous study on dopaminergic neuron-like cells treated with cocaine^27^.

Some of the miRNAs reported in the present study had also been found down-regulated by cocaine in previous works. For example, miR-124 is down-regulated in the NAc of mice chronically exposed to cocaine^49^ and in neuronal cell cultures (Be(2)-M17 and SH-SY5Y) treated with cocaine^50^. Moreover, overexpression of this gene in NAc attenuates cocaine-induced place preference in mice^51^. On the other hand, miR-137 and miR-101 are down-regulated in the NAc of cocaine self-administrated rats compared to controls^52^. This is the first time that miR-9-5p, miR-369-3p and miR-153-3p are found down-regulated after cocaine treatment. MiR-9 is one of the most highly expressed miRNAs in the adult vertebrate brain and it plays an important role in brain development, neurogenesis, synaptic plasticity and memory^53–57^. Furthermore, a recent study associated fetal alcohol exposure with an increase of miR-9 in the pituitary, which represses the *D2r* gene and its spliced variant *D2s*^58^. The miR-369 is located in the miR379–410 cluster, which contains 38 miRNAs involved in neuronal development and function^59^. MiR-369-3p regulates the expression of *Ncad*, *Adam19*, and *TrappC8*, very important in neurogenesis and neuronal migration^60^. Finally, miR-153 inhibits *Cacna1c* to suppress neuroendocrine secretion (insulin and dopamine)^61^.

Interestingly, three of the genes encoding differentially expressed mature miRNAs (hsa-miR-9-1, hsa-miR-153-1 and hsa-miR-124-3) were found to be nominally associated with cocaine dependence in this study. Previous studies had reported genes which expression is altered by cocaine and that also carry risk variants for cocaine dependence, or viceversa (*NFAT5, PLCB1* and *NTNG1*)^27,62,63^. The findings in the present study, together with the previous ones, support the hypothesis that genes up-regulated or down-regulated by cocaine may also contribute to the susceptibility to cocaine dependence.

Strengths and limitations of the present study should be discussed. Our study was performed in a dopaminergic neuron-like model, from a tumor cell line, which performance may differ from the events that take place in the brain. However, we were able to detect previously described miRNAs that had shown down-regulation by cocaine in mice, and three new miRNAs. On the other hand, three genes encoding these altered miRNAs were found to carry genetic variants nominally associated with cocaine dependence in human samples, despite the relatively small sample size of the meta-analysis used. Increasing the number of individuals of this study may increase the strength of the associations. Finally, it is important to note that, although many tools/databases are available to predict mRNA:miRNA interactions, the overlap between the outputs is often very low^17^, so it is important to validate them. Here, we demonstrated that WebGestalt is a very useful tool to identify enrichment of miRNA-binding sites in a list of potential target genes, since we could validate changes in the expression of 7 out of 11 predicted miRNAs. This strategy was previously used by others with a similar performance^64,65^. Further studies are needed to validate the mRNA:miRNA interactions identified.

In conclusion, we found seven miRNAs down-regulated in dopaminergic neuron-like cells after an acute cocaine treatment, which might regulate several protein-coding genes previously reported to be up-regulated in this model. Here we highlighted new miRNAs that may be involved in cocaine-induced changes of gene expression. Furthermore, using a GWAS meta-analysis of clinical samples, we found association between cocaine dependence and three genes encoding these differentially expressed miRNAs. All these results support the idea that genes which expression is altered by cocaine might play an important role in the susceptibility to cocaine dependence, and also that miRNAs may be relevant players in modulating cocaine effects and dependence.

## Electronic supplementary material

Supplementary Table 1

Supplementary Table 2
