# Supplementary material for: MiR-9, miR-153 and miR-124 are down-regulated by acute exposure to cocaine in a dopaminergic cell model and may contribute to cocaine dependence
Source: Transl Psychiatry. 2018 Aug 30;8:173. doi: 10.1038/s41398-018-0224-5 (PMC6117282; doi:10.1038/s41398-018-0224-5)
Supplement: Supplementary file 1 — Supplementary Table 1 [file 41398_2018_224_MOESM1_ESM.docx]

| **Supplementary table 1.** List of predicted miRNAs targeting the differentially expressed genes. | | | | |  |  |
| --- | --- | --- | --- | --- | --- | --- |
|  | |  |  |  |  |  |
| **microRNA** | **miRNA seed** | **Number of genes*** | **EntrezGene** | **Statistics** | ***p-value*^#^** | **Adj *p-value*^&^** |
| **Down-regulated target genes** | |  |  |  |  |  |
|  |  |  |  |  |  |  |
| MIR-320 | CAGCTTT | [15](file:///C:\Users\TEMP\Rar$EXa0.490\miRNA1446572694\final_mirna_geneset_file_1446572694.html#hsa_CAGCTTT,MIR-320) | 1627 5756 27125 9868 140775 5157123264 23162 22859 7534 80031 549522907 9403 7037 | C=243; O=15; E=4.07; R=3.68 | 1.62E-05 | 1.50E-03 |
| MIR-200B,MIR-200C, MIR-429 | CAGTATT | [21](file:///C:\Users\TEMP\Rar$EXa0.490\miRNA1446572694\final_mirna_geneset_file_1446572694.html#hsa_CAGTATT,MIR-200B,MIR-200C,MIR-429) | 2626 4774 80031 8491 121441 78135495 9669 11168 57589 3953 5461929994 23164 22858 56965 26190 73217917 846 6048 | C=453; O=21; E=7.59; R=2.77 | 2.87E-05 | 1.50E-03 |
| MIR-496 | CATGTAA | [12](file:///C:\Users\TEMP\Rar$EXa0.490\miRNA1446572694\final_mirna_geneset_file_1446572694.html#hsa_CATGTAA,MIR-496) | 9868 4335 29072 7095 9589 8003110605 1031 1810 9678 7534 7750 | C=169; O=12; E=2.83; R=4.24 | 2.92E-05 | 1.50E-03 |
| MIR-412 | GGTGAAG | [6](file:///C:\Users\TEMP\Rar$EXa0.490\miRNA1446572694\final_mirna_geneset_file_1446572694.html#hsa_GGTGAAG,MIR-412) | 1184 2309 7812 92305 5500 8720 | C=58; O=6; E=0.97; R=6.17 | 4.00E-04 | 0.0151 |
| MIR-124A | TGCCTTA | [20](file:///C:\Users\TEMP\Rar$EXa0.490\miRNA1446572694\final_mirna_geneset_file_1446572694.html#hsa_TGCCTTA,MIR-124A) | 55830 60481 10160 10424 23762 2608851143 26994 80031 5870 5663 464127125 8763 29994 92305 9441 1129396509 5911 | C=535; O=20; E=8.97; R=2.23 | 8.00E-04 | 0.0201 |
| MIR-218 | AAGCACA | [16](file:///C:\Users\TEMP\Rar$EXa0.490\miRNA1446572694\final_mirna_geneset_file_1446572694.html#hsa_AAGCACA,MIR-218) | 1627 60481 1277 51143 23162 5870140730 131566 7812 27123 8089 22858112939 5524 22859 5500 | C=382; O=16; E=6.40; R=2.50 | 8.00E-04 | 0.0201 |
|  |  |  |  |  |  |  |
| **Up-regulated target genes** | |  |  |  |  |  |
|  | |  |  |  |  |  |
| MIR-202 | ATAGGAA | [15](file:///C:\Users\TEMP\Rar$EXa0.573\miRNA1446574303\final_mirna_geneset_file_1446574303.html#hsa_ATAGGAA,MIR-202) | 7798 80155 1997 9887 23389 55740 59338 867 58516 4297 5469 29843 55704 10513 6421 | C=100; O=15; E=1.93; R=7.78 | 8.19E-10 | 1.48E-07 |
| MIR-17-5P,MIR-20A, MIR-106A,MIR-106B, MIR-20B,MIR-519D | GCACTTT | [32](file:///C:\Users\TEMP\Rar$EXa0.573\miRNA1446574303\final_mirna_geneset_file_1446574303.html#hsa_GCACTTT,MIR-17-5P,MIR-20A,MIR-106A,MIR-106B,MIR-20B,MIR-519D) | 23040 7798 659 55284 80829 10618 4090 80143 5934 10725 10681 7126 800 51465 392 168850 29843 7091 80821 83737 55074 11214 6659 58508 22862 222236 2909 29028 10000 9644 58516 51277 | C=574; O=32; E=11.07; R=2.89 | 8.42E-08 | 7.62E-06 |
| MIR-93,MIR-302A, MIR-302B,MIR-302C, MIR-302D,MIR-372, MIR-373,MIR-520E, MIR-520A,MIR-526B, MIR-520B,MIR-520C, MIR-520D | AGCACTT | [22](file:///C:\Users\TEMP\Rar$EXa0.573\miRNA1446574303\final_mirna_geneset_file_1446574303.html#hsa_AGCACTT,MIR-93,MIR-302A,MIR-302B,MIR-302C,MIR-302D,MIR-372,MIR-373,MIR-520E,MIR-520A,MIR-526B,MIR-520B,MIR-520C,MIR-520D) | 23040 55284 80829 10681 7126 7586 51465 4297 168850 29843 7091 80821 10771 55074 58508 22862 222236 4087 29028 81493 51277 58528 | C=322; O=22; E=6.21; R=3.54 | 3.31E-07 | 2.00E-05 |
| MIR-30A-3P,MIR-30E-3P | ACTGAAA | [16](file:///C:\Users\TEMP\Rar$EXa0.573\miRNA1446574303\final_mirna_geneset_file_1446574303.html#hsa_ACTGAAA,MIR-30A-3P,MIR-30E-3P) | 23040 9522 2589 51465 7398 7150 134553 7570 79982 867 1184 8028 83439 6263 51277 5892 | C=193; O=16; E=3.72; R=4.30 | 1.12E-06 | 4.34E-05 |
| **MIR-181A** | **TGAATGT** | [**26**](file:///C:\Users\TEMP\Rar$EXa0.573\miRNA1446574303\final_mirna_geneset_file_1446574303.html#hsa_TGAATGT,MIR-181A,MIR-181B,MIR-181C,MIR-181D) | **23321 659 2186 26118 80155 55740 3267 10725 8065 8503 55137 168850 29843 9877 3609 9774 54842 22862 23022 6249 10000 8473 81688 64062 10611 5885** | **C=462; O=26; E=8.91; R=2.92** | **1.20E-06** | **4.34E-05** |
| MIR-200B,MIR-200C,MIR-429 | CAGTATT | [25](file:///C:\Users\TEMP\Rar$EXa0.573\miRNA1446574303\final_mirna_geneset_file_1446574303.html#hsa_CAGTATT,MIR-200B,MIR-200C,MIR-429) | 4008 23321 9522 2186 55284 2119 331 3267 22846 55854 5514 6935 3184 7003 55074 9669 51199 6249 9857 8473 51719 81688 23671 9644 151987 | C=453; O=25; E=8.73; R=2.86 | 2.74E-06 | 8.27E-05 |
| MIR-25,MIR-32,MIR-92,MIR-363,MIR-367 | GTGCAAT | [19](file:///C:\Users\TEMP\Rar$EXa0.573\miRNA1446574303\final_mirna_geneset_file_1446574303.html#hsa_GTGCAAT,MIR-25,MIR-32,MIR-92,MIR-363,MIR-367) | 23077 7798 659 55284 7204 10725 8503 96459 1130 7003 59338 6659 51290 8473 81688 3685 7029 9644 5885 | C=298; O=19; E=5.74; R=3.31 | 5.80E-06 | 1.00E-04 |
| MIR-199A | CTACTGT | [14](file:///C:\Users\TEMP\Rar$EXa0.573\miRNA1446574303\final_mirna_geneset_file_1446574303.html#hsa_CTACTGT,MIR-199A) | 26118 80155 5934 4154 6935 9728 7150 27332 80267 867 27436 58517 10000 81688 | C=176; O=14; E=3.39; R=4.13 | 8.44E-06 | 2.00E-04 |
| MIR-130A,MIR-301, MIR-130B | TTGCACT | [22](file:///C:\Users\TEMP\Rar$EXa0.573\miRNA1446574303\final_mirna_geneset_file_1446574303.html#hsa_TTGCACT,MIR-130A,MIR-301,MIR-130B) | 23321 659 2186 80829 4090 55082 3267 4154 392 4297 168850 55704 91452 9749 6659 54842 1184 23060 8028 2909 6249 8473 | C=397; O=22; E=7.65; R=2.87 | 1.02E-05 | 2.00E-04 |
| MIR-129 | GCAAAAA | [14](file:///C:\Users\TEMP\Rar$EXa0.573\miRNA1446574303\final_mirna_geneset_file_1446574303.html#hsa_GCAAAAA,MIR-129) | 23040 659 2186 7204 23389 56853 2589 55137 55074 3609 23760 6659 23060 10611 | C=183; O=14; E=3.53; R=3.97 | 1.32E-05 | 2.00E-04 |
| MIR-29A,MIR-29B, MIR-29C | TGGTGCT | [25](file:///C:\Users\TEMP\Rar$EXa0.573\miRNA1446574303\final_mirna_geneset_file_1446574303.html#hsa_TGGTGCT,MIR-29A,MIR-29B,MIR-29C) | 7798 5565 80829 9643 10725 57590 7126 8503 10116 4297 29843 6421 134553 55074 59338 11214 27436 23060 79915 6599 10000 57542 7528 9644 254065 | C=503; O=25; E=9.70; R=2.58 | 1.67E-05 | 3.00E-04 |
| MIR-204,MIR-211 | AAAGGGA | [15](file:///C:\Users\TEMP\Rar$EXa0.573\miRNA1446574303\final_mirna_geneset_file_1446574303.html#hsa_AAAGGGA,MIR-204,MIR-211) | 23451 80829 26118 80155 23389 55082 4154 96459 4297 7430 6659 83439 6249 7799 6749 | C=216; O=15; E=4.16; R=3.60 | 2.07E-05 | 3.00E-04 |
| MIR-19A,MIR-19B | TTTGCAC | [25](file:///C:\Users\TEMP\Rar$EXa0.573\miRNA1446574303\final_mirna_geneset_file_1446574303.html#hsa_TTTGCAC,MIR-19A,MIR-19B) | 659 2186 80829 23621 23389 10618 4090 55082 4154 8503 392 168850 55704 91452 23215 10771 6659 54842 22862 23060 6249 9857 8473 11127 51719 | C=501; O=25; E=9.66; R=2.59 | 1.56E-05 | 3.00E-04 |
| **MIR-369-3P** | **GTATTAT** | [**14**](file:///C:\Users\TEMP\Rar$EXa0.573\miRNA1446574303\final_mirna_geneset_file_1446574303.html#hsa_GTATTAT,MIR-369-3P) | **10808 659 23621 26118 2119 4154 96459 6935 4297 7091 6433 55074 6659 64062** | **C=196; O=14; E=3.78; R=3.71** | **2.85E-05** | **4.00E-04** |
| MIR-142-3P | ACACTAC | [11](file:///C:\Users\TEMP\Rar$EXa0.573\miRNA1446574303\final_mirna_geneset_file_1446574303.html#hsa_ACACTAC,MIR-142-3P) | 25831 5862 9643 29123 4297 80267 9774 22862 27436 3685 254065 | C=128; O=11; E=2.47; R=4.46 | 3.84E-05 | 5.00E-04 |
| MIR-330 | TGCTTTG | [18](file:///C:\Users\TEMP\Rar$EXa0.573\miRNA1446574303\final_mirna_geneset_file_1446574303.html#hsa_TGCTTTG,MIR-330) | 7798 659 331 9887 4154 10643 168850 134553 83737 64746 7003 1184 2260 8028 8473 10611 9644 9818 | C=319; O=18; E=6.15; R=2.93 | 5.17E-05 | 6.00E-04 |
| **MIR-137** | **AAGCAAT** | [**14**](file:///C:\Users\TEMP\Rar$EXa0.573\miRNA1446574303\final_mirna_geneset_file_1446574303.html#hsa_AAGCAAT,MIR-137) | **659 26118 4297 5469 7091 10771 55074 5339 22862 6651 5903 8473 64062 81493** | **C=212; O=14; E=4.09; R=3.43** | **6.72E-05** | **7.00E-04** |
| MIR-520D | TTTGTAG | [18](file:///C:\Users\TEMP\Rar$EXa0.573\miRNA1446574303\final_mirna_geneset_file_1446574303.html#hsa_TTTGTAG,MIR-520D) | 10163 55284 2119 50810 55740 55082 10643 1997 7003 80267 7570 6651 8028 10521 5903 9857 7799 6789 | C=330; O=18; E=6.36; R=2.83 | 7.98E-05 | 8.00E-04 |
| MIR-519C,MIR-519B, MIR-519A | TGCACTT | [21](file:///C:\Users\TEMP\Rar$EXa0.573\miRNA1446574303\final_mirna_geneset_file_1446574303.html#hsa_TGCACTT,MIR-519C,MIR-519B,MIR-519A) | 659 80829 4090 4154 10725 10643 5514 800 392 55704 83737 7003 11214 6659 54842 2909 10000 6263 8473 9644 58516 | C=434; O=21; E=8.37; R=2.51 | 1.00E-04 | 9.00E-04 |
| MIR-15A,MIR-16, MIR-15B,MIR-195, MIR-424,MIR-497 | TGCTGCT | [25](file:///C:\Users\TEMP\Rar$EXa0.573\miRNA1446574303\final_mirna_geneset_file_1446574303.html#hsa_TGCTGCT,MIR-15A,MIR-16,MIR-15B,MIR-195,MIR-424,MIR-497) | 23040 23321 7798 23621 55740 4090 22808 7586 51465 4297 26135 1130 7091 206358 6645 59338 1184 6651 23060 10000 8473 51719 10611 58516 55161 | C=574; O=25; E=11.07; R=2.26 | 1.00E-04 | 9.00E-04 |
| **MIR-9** | **ACCAAAG** | [**22**](file:///C:\Users\TEMP\Rar$EXa0.573\miRNA1446574303\final_mirna_geneset_file_1446574303.html#hsa_ACCAAAG,MIR-9) | **23321 23621 56853 10618 3267 4154 10643 7586 1997 8503 96459 29843 10645 80267 9774 6651 25778 10521 5903 4750 9857 57542** | **C=489; O=22; E=9.43; R=2.33** | **2.00E-04** | **1.60E-03** |
| MIR-520F | AAGCACT | [14](file:///C:\Users\TEMP\Rar$EXa0.573\miRNA1446574303\final_mirna_geneset_file_1446574303.html#hsa_AAGCACT,MIR-520F) | 80829 7586 7126 55137 4297 29843 168850 80821 22862 222236 29028 9857 81493 127933 | C=236; O=14; E=4.55; R=3.08 | 2.00E-04 | 1.60E-03 |
| **MIR-124A** | **TGCCTTA** | [**23**](file:///C:\Users\TEMP\Rar$EXa0.573\miRNA1446574303\final_mirna_geneset_file_1446574303.html#hsa_TGCCTTA,MIR-124A) | **26088 23621 80155 55082 345557 57590 8065 55752 9331 134553 9733 7003 133619 5411 23022 9857 10000 6263 8473 81493 7029 9818 6789** | **C=535; O=23; E=10.31; R=2.23** | **3.00E-04** | **2.10E-03** |
| MIR-200A | CAGTGTT | [16](file:///C:\Users\TEMP\Rar$EXa0.573\miRNA1446574303\final_mirna_geneset_file_1446574303.html#hsa_CAGTGTT,MIR-141,MIR-200A) | 23040 23621 26118 23389 22809 4154 3725 7150 6421 23271 23760 867 6651 23060 8473 11127 | C=304; O=16; E=5.86; R=2.73 | 3.00E-04 | 2.10E-03 |
| MIR-30A-5P,MIR-30C, MIR-30D,MIR-30B, MIR-30E-5P | TGTTTAC | [24](file:///C:\Users\TEMP\Rar$EXa0.573\miRNA1446574303\final_mirna_geneset_file_1446574303.html#hsa_TGTTTAC,MIR-30A-5P,MIR-30C,MIR-30D,MIR-30B,MIR-30E-5P) | 9522 29855 26057 7072 345557 10725 2589 51465 4297 1130 8819 9331 10645 206358 80267 9749 5411 6659 54842 22862 6651 25778 27436 9644 | C=567; O=24; E=10.93; R=2.20 | 3.00E-04 | 2.10E-03 |
| MIR-524 | CTTTGTA | [20](file:///C:\Users\TEMP\Rar$EXa0.573\miRNA1446574303\final_mirna_geneset_file_1446574303.html#hsa_CTTTGTA,MIR-524) | 10163 57634 25831 80829 80155 3725 10265 7126 219333 7091 80821 7003 6924 6659 6651 23060 55677 7528 58516 9818 | C=427; O=20; E=8.23; R=2.43 | 3.00E-04 | 2.10E-03 |
| MIR-18A,MIR-18B | GCACCTT | [9](file:///C:\Users\TEMP\Rar$EXa0.573\miRNA1446574303\final_mirna_geneset_file_1446574303.html#hsa_GCACCTT,MIR-18A,MIR-18B) | 23321 26118 10725 55704 23271 6651 4087 254065 6789 | C=114; O=9; E=2.20; R=4.10 | 4.00E-04 | 2.70E-03 |
| **MIR-186** | **ATTCTTT** | [**14**](file:///C:\Users\TEMP\Rar$EXa0.573\miRNA1446574303\final_mirna_geneset_file_1446574303.html#hsa_ATTCTTT,MIR-186) | **23040 331 80155 55082 4154 10725 134553 9877 5411 8507 22862 7175 64062 7528** | **C=259; O=14; E=4.99; R=2.80** | **5.00E-04** | **3.10E-03** |
| MIR-512-3P | CAGCACT | [10](file:///C:\Users\TEMP\Rar$EXa0.573\miRNA1446574303\final_mirna_geneset_file_1446574303.html#hsa_CAGCACT,MIR-512-3P) | 23040 23321 80829 331 4154 4297 168850 58508 4087 7029 | C=146; O=10; E=2.81; R=3.55 | 5.00E-04 | 3.10E-03 |
| MIR-381 | CTTGTAT | [12](file:///C:\Users\TEMP\Rar$EXa0.573\miRNA1446574303\final_mirna_geneset_file_1446574303.html#hsa_CTTGTAT,MIR-381) | 29855 23389 5862 55082 10513 55752 1108 83737 10771 6659 51199 10492 | C=201; O=12; E=3.87; R=3.10 | 6.00E-04 | 3.40E-03 |
| MIR-513 | CCTGTGA | [9](file:///C:\Users\TEMP\Rar$EXa0.573\miRNA1446574303\final_mirna_geneset_file_1446574303.html#hsa_CCTGTGA,MIR-513) | 9887 55737 8555 84922 23524 7091 8507 2260 8473 | C=121; O=9; E=2.33; R=3.86 | 6.00E-04 | 3.40E-03 |
| MIR-188 | AAGGGAT | [7](file:///C:\Users\TEMP\Rar$EXa0.573\miRNA1446574303\final_mirna_geneset_file_1446574303.html#hsa_AAGGGAT,MIR-188) | 7798 80829 4154 96459 3609 9749 6659 | C=74; O=7; E=1.43; R=4.91 | 6.00E-04 | 3.40E-03 |
| MIR-380-3P | ATTACAT | [8](file:///C:\Users\TEMP\Rar$EXa0.573\miRNA1446574303\final_mirna_geneset_file_1446574303.html#hsa_ATTACAT,MIR-380-3P) | 55082 4154 8065 55137 168850 22862 64062 3685 | C=100; O=8; E=1.93; R=4.15 | 7.00E-04 | 3.80E-03 |
| **MIR-153** | **CTATGCA** | [**12**](file:///C:\Users\TEMP\Rar$EXa0.573\miRNA1446574303\final_mirna_geneset_file_1446574303.html#hsa_CTATGCA,MIR-153) | **659 2186 55284 55752 6421 10645 1184 23060 6263 7029 7799 9818** | **C=211; O=12; E=4.07; R=2.95** | **8.00E-04** | **4.30E-03** |
| MIR-199A,MIR-199B | ACACTGG | [10](file:///C:\Users\TEMP\Rar$EXa0.573\miRNA1446574303\final_mirna_geneset_file_1446574303.html#hsa_ACACTGG,MIR-199A,MIR-199B) | 80829 55082 10513 867 9669 5903 6249 28996 9644 6789 | C=155; O=10; E=2.99; R=3.35 | 9.00E-04 | 4.40E-03 |
| MIR-205 | ATGAAGG | [10](file:///C:\Users\TEMP\Rar$EXa0.573\miRNA1446574303\final_mirna_geneset_file_1446574303.html#hsa_ATGAAGG,MIR-205) | 2186 9887 10725 55752 5469 168850 9331 7003 23271 23060 | C=155; O=10; E=2.99; R=3.35 | 9.00E-04 | 4.40E-03 |
| MIR-432 | CTCCAAG | [7](file:///C:\Users\TEMP\Rar$EXa0.573\miRNA1446574303\final_mirna_geneset_file_1446574303.html#hsa_CTCCAAG,MIR-432) | 7798 80829 23621 10725 9878 27436 10611 | C=80; O=7; E=1.54; R=4.54 | 9.00E-04 | 4.40E-03 |
| MIR-493 | ATGTACA | [15](file:///C:\Users\TEMP\Rar$EXa0.573\miRNA1446574303\final_mirna_geneset_file_1446574303.html#hsa_ATGTACA,MIR-493) | 26057 23389 55740 51429 3725 8065 5514 96459 55137 168850 7091 54842 8028 29028 23196 | C=307; O=15; E=5.92; R=2.53 | 1.00E-03 | 4.80E-03 |
| MIR-346 | GGCAGAC | [5](file:///C:\Users\TEMP\Rar$EXa0.573\miRNA1446574303\final_mirna_geneset_file_1446574303.html#hsa_GGCAGAC,MIR-346) | 4154 8503 7536 23060 8473 | C=41; O=5; E=0.79; R=6.33 | 1.10E-03 | 5.10E-03 |
| **MIR-101** | **GTACTGT** | [**13**](file:///C:\Users\TEMP\Rar$EXa0.573\miRNA1446574303\final_mirna_geneset_file_1446574303.html#hsa_GTACTGT,MIR-101) | **26057 26118 2119 4154 29123 4297 343099 80267 867 25778 8473 9644 6789** | **C=252; O=13; E=4.86; R=2.68** | **1.30E-03** | **5.90E-03** |
| MIR-433 | ATCATGA | [8](file:///C:\Users\TEMP\Rar$EXa0.573\miRNA1446574303\final_mirna_geneset_file_1446574303.html#hsa_ATCATGA,MIR-433) | 9522 2186 7072 2589 7150 4943 6249 55161 | C=111; O=8; E=2.14; R=3.74 | 1.40E-03 | 6.20E-03 |
| **MIR-105** | **GCATTTG** | [**10**](file:///C:\Users\TEMP\Rar$EXa0.573\miRNA1446574303\final_mirna_geneset_file_1446574303.html#hsa_GCATTTG,MIR-105) | **5862 8082 9643 4297 8861 22862 6651 8473 7799 23196** | **C=167; O=10; E=3.22; R=3.11** | **1.50E-03** | **6.30E-03** |
| MIR-448 | ATATGCA | [11](file:///C:\Users\TEMP\Rar$EXa0.573\miRNA1446574303\final_mirna_geneset_file_1446574303.html#hsa_ATATGCA,MIR-448) | 5565 55284 23451 23389 10513 1130 26135 1184 5903 64062 6789 | C=196; O=11; E=3.78; R=2.91 | 1.50E-03 | 6.30E-03 |
| MIR-214 | CCTGCTG | [12](file:///C:\Users\TEMP\Rar$EXa0.573\miRNA1446574303\final_mirna_geneset_file_1446574303.html#hsa_CCTGCTG,MIR-214) | 7798 23621 80155 10618 83737 6433 4943 55074 867 23060 8028 6599 | C=228; O=12; E=4.40; R=2.73 | 1.60E-03 | 6.60E-03 |
| **MIR-9** | **TAGCTTT** | [**12**](file:///C:\Users\TEMP\Rar$EXa0.573\miRNA1446574303\final_mirna_geneset_file_1446574303.html#hsa_TAGCTTT,MIR-9) | **55284 55740 51773 3267 800 29843 10771 7021 7003 6659 10000 6789** | **C=231; O=12; E=4.45; R=2.69** | **1.80E-03** | **7.10E-03** |
| MIR-302C | ATGTTAA | [12](file:///C:\Users\TEMP\Rar$EXa0.573\miRNA1446574303\final_mirna_geneset_file_1446574303.html#hsa_ATGTTAA,MIR-302C) | 23451 4154 10725 23008 7398 64746 7003 9749 6651 7528 23196 55161 | C=231; O=12; E=4.45; R=2.69 | 1.80E-03 | 7.10E-03 |
| MIR-26A,MIR-26B | TACTTGA | [14](file:///C:\Users\TEMP\Rar$EXa0.573\miRNA1446574303\final_mirna_geneset_file_1446574303.html#hsa_TACTTGA,MIR-26A,MIR-26B) | 57634 80155 23389 22808 96459 26135 91452 8507 7915 9857 57542 9644 23196 55161 | C=295; O=14; E=5.69; R=2.46 | 1.90E-03 | 7.30E-03 |
| MIR-518A-2 | TTTGCAG | [11](file:///C:\Users\TEMP\Rar$EXa0.573\miRNA1446574303\final_mirna_geneset_file_1446574303.html#hsa_TTTGCAG,MIR-518A-2) | 26057 25831 5565 331 23389 3267 7536 83737 5411 58508 58517 | C=204; O=11; E=3.93; R=2.80 | 2.10E-03 | 7.90E-03 |
| MIR-324-5P | GGGATGC | [5](file:///C:\Users\TEMP\Rar$EXa0.573\miRNA1446574303\final_mirna_geneset_file_1446574303.html#hsa_GGGATGC,MIR-324-5P) | 80829 10725 8065 54856 56987 | C=48; O=5; E=0.93; R=5.40 | 2.20E-03 | 8.00E-03 |
| MIR-506 | GTGCCTT | [25](file:///C:\Users\TEMP\Rar$EXa0.573\miRNA1446574303\final_mirna_geneset_file_1446574303.html#hsa_GTGCCTT,MIR-506) | 29855 26118 80155 55740 4090 345557 10725 55737 8555 7398 4297 7091 3030 7003 5339 79982 134492 867 23022 23060 6263 11127 9644 7799 6789 | C=696; O=25; E=13.42; R=1.86 | 2.20E-03 | 8.00E-03 |
| MIR-10A,MIR-10B | ACAGGGT | [8](file:///C:\Users\TEMP\Rar$EXa0.573\miRNA1446574303\final_mirna_geneset_file_1446574303.html#hsa_ACAGGGT,MIR-10A,MIR-10B) | 23321 80155 10725 10513 2589 55137 10771 6651 | C=120; O=8; E=2.31; R=3.46 | 2.30E-03 | 8.20E-03 |
| MIR-499 | AGTCTTA | [6](file:///C:\Users\TEMP\Rar$EXa0.573\miRNA1446574303\final_mirna_geneset_file_1446574303.html#hsa_AGTCTTA,MIR-499) | 29855 55082 7150 27436 57542 58516 | C=73; O=6; E=1.41; R=4.26 | 2.80E-03 | 9.40E-03 |
| MIR-23A,MIR-23B | AATGTGA | [17](file:///C:\Users\TEMP\Rar$EXa0.573\miRNA1446574303\final_mirna_geneset_file_1446574303.html#hsa_AATGTGA,MIR-23A,MIR-23B) | 26118 331 80155 345557 6935 96459 64388 7150 94104 7003 9749 23022 51199 58517 51719 9644 6789 | C=412; O=17; E=7.94; R=2.14 | 2.80E-03 | 9.40E-03 |
| MIR-320 | CAGCTTT | [12](file:///C:\Users\TEMP\Rar$EXa0.573\miRNA1446574303\final_mirna_geneset_file_1446574303.html#hsa_CAGCTTT,MIR-320) | 80829 9887 55740 10643 800 26135 5927 23271 23022 6651 8473 51719 | C=243; O=12; E=4.68; R=2.56 | 2.80E-03 | 9.40E-03 |
| MIR-520A,MIR-525 | CTCTGGA | [9](file:///C:\Users\TEMP\Rar$EXa0.573\miRNA1446574303\final_mirna_geneset_file_1446574303.html#hsa_CTCTGGA,MIR-520A,MIR-525) | 80829 2119 80155 4297 6651 83439 8473 58516 9818 | C=153; O=9; E=2.95; R=3.05 | 2.90E-03 | 9.50E-03 |
| MIR-133A,MIR-133B | GGGACCA | [10](file:///C:\Users\TEMP\Rar$EXa0.573\miRNA1446574303\final_mirna_geneset_file_1446574303.html#hsa_GGGACCA,MIR-133A,MIR-133B) | 23077 23321 80829 56853 10725 9728 29843 6433 867 5903 | C=186; O=10; E=3.59; R=2.79 | 3.40E-03 | 0.011 |
| MIR-520G,MIR-520H | CACTTTG | [11](file:///C:\Users\TEMP\Rar$EXa0.573\miRNA1446574303\final_mirna_geneset_file_1446574303.html#hsa_CACTTTG,MIR-520G,MIR-520H) | 55284 80829 10618 7126 29843 219333 7091 8507 22862 51199 58516 | C=220; O=11; E=4.24; R=2.59 | 3.70E-03 | 0.0117 |
| MIR-144 | ATACTGT | [10](file:///C:\Users\TEMP\Rar$EXa0.573\miRNA1446574303\final_mirna_geneset_file_1446574303.html#hsa_ATACTGT,MIR-144) | 7204 80155 10513 168850 55704 64746 22862 6651 51199 58516 | C=195; O=10; E=3.76; R=2.66 | 4.70E-03 | 0.0147 |
| MIR-511 | AAAGACA | [10](file:///C:\Users\TEMP\Rar$EXa0.573\miRNA1446574303\final_mirna_geneset_file_1446574303.html#hsa_AAAGACA,MIR-511) | 23321 5862 10643 8503 80267 27436 9857 64062 7528 58516 | C=197; O=10; E=3.80; R=2.63 | 5.00E-03 | 0.0153 |
| MIR-376A,MIR-376B | TCTATGA | [6](file:///C:\Users\TEMP\Rar$EXa0.573\miRNA1446574303\final_mirna_geneset_file_1446574303.html#hsa_TCTATGA,MIR-376A,MIR-376B) | 2186 56853 55740 5514 10771 9818 | C=83; O=6; E=1.60; R=3.75 | 5.40E-03 | 0.0163 |
| MIR-338 | ATGCTGG | [7](file:///C:\Users\TEMP\Rar$EXa0.573\miRNA1446574303\final_mirna_geneset_file_1446574303.html#hsa_ATGCTGG,MIR-338) | 23077 26088 26118 55082 9643 9728 23196 | C=111; O=7; E=2.14; R=3.27 | 5.70E-03 | 0.0166 |
| MIR-496 | CATGTAA | [9](file:///C:\Users\TEMP\Rar$EXa0.573\miRNA1446574303\final_mirna_geneset_file_1446574303.html#hsa_CATGTAA,MIR-496) | 23040 55284 7072 55186 55752 9331 79982 55161 5885 | C=169; O=9; E=3.26; R=2.76 | 5.60E-03 | 0.0166 |
| MIR-409-3P | AACATTC | [8](file:///C:\Users\TEMP\Rar$EXa0.573\miRNA1446574303\final_mirna_geneset_file_1446574303.html#hsa_AACATTC,MIR-409-3P) | 51773 3725 51465 168850 1130 80267 8473 51495 | C=140; O=8; E=2.70; R=2.96 | 5.80E-03 | 0.0167 |
| LET-7A,LET-7B,LET-7C, LET-7D,LET-7E,LET-7F, MIR-98,LET-7G,LET-7I | CTACCTC | [15](file:///C:\Users\TEMP\Rar$EXa0.573\miRNA1446574303\final_mirna_geneset_file_1446574303.html#hsa_CTACCTC,LET-7A,LET-7B,LET-7C,LET-7D,LET-7E,LET-7F,MIR-98,LET-7G,LET-7I) | 345557 10643 2589 96459 1108 55137 6433 80267 867 22862 222236 5903 6599 7799 8161 | C=379; O=15; E=7.31; R=2.05 | 7.10E-03 | 0.0201 |
| MIR-495 | GTTTGTT | [11](file:///C:\Users\TEMP\Rar$EXa0.573\miRNA1446574303\final_mirna_geneset_file_1446574303.html#hsa_GTTTGTT,MIR-495) | 23040 29855 9643 10725 10513 3725 3609 5411 867 27436 8473 | C=243; O=11; E=4.68; R=2.35 | 7.80E-03 | 0.0217 |
| MIR-370 | CAGCAGG | [8](file:///C:\Users\TEMP\Rar$EXa0.573\miRNA1446574303\final_mirna_geneset_file_1446574303.html#hsa_CAGCAGG,MIR-370) | 80155 9887 4297 84914 10771 3609 8473 373863 | C=150; O=8; E=2.89; R=2.77 | 8.70E-03 | 0.0238 |
| MIR-324-3P | GGCAGTG | [6](file:///C:\Users\TEMP\Rar$EXa0.573\miRNA1446574303\final_mirna_geneset_file_1446574303.html#hsa_GGCAGTG,MIR-324-3P) | 10163 26118 2119 23008 6421 7150 | C=92; O=6; E=1.77; R=3.38 | 8.80E-03 | 0.0238 |
| MIR-342 | GTGTGAG | [5](file:///C:\Users\TEMP\Rar$EXa0.573\miRNA1446574303\final_mirna_geneset_file_1446574303.html#hsa_GTGTGAG,MIR-342) | 659 29855 4297 10645 7029 | C=67; O=5; E=1.29; R=3.87 | 9.40E-03 | 0.025 |
| MIR-522 | ACCATTT | [8](file:///C:\Users\TEMP\Rar$EXa0.573\miRNA1446574303\final_mirna_geneset_file_1446574303.html#hsa_ACCATTT,MIR-522) | 23077 4154 3725 51465 7150 9877 8507 10492 | C=155; O=8; E=2.99; R=2.68 | 0.0105 | 0.0275 |
| MIR-151 | AGTCTAG | [3](file:///C:\Users\TEMP\Rar$EXa0.573\miRNA1446574303\final_mirna_geneset_file_1446574303.html#hsa_AGTCTAG,MIR-151) | 7419 55752 51290 | C=24; O=3; E=0.46; R=6.48 | 0.0107 | 0.0277 |
| MIR-517 | TCTAGAG | [4](file:///C:\Users\TEMP\Rar$EXa0.573\miRNA1446574303\final_mirna_geneset_file_1446574303.html#hsa_TCTAGAG,MIR-517) | 55082 9728 25820 3609 | C=46; O=4; E=0.89; R=4.51 | 0.0117 | 0.0298 |
| MIR-221,MIR-222 | ATGTAGC | [7](file:///C:\Users\TEMP\Rar$EXa0.573\miRNA1446574303\final_mirna_geneset_file_1446574303.html#hsa_ATGTAGC,MIR-221,MIR-222) | 3267 22846 10265 51465 4297 22862 8473 | C=129; O=7; E=2.49; R=2.81 | 0.0126 | 0.0317 |
| MIR-148A,MIR-152,MIR-148B | TGCACTG | [12](file:///C:\Users\TEMP\Rar$EXa0.573\miRNA1446574303\final_mirna_geneset_file_1446574303.html#hsa_TGCACTG,MIR-148A,MIR-152,MIR-148B) | 55284 2119 80155 55082 3267 8065 8503 4297 7003 9749 8028 81688 | C=298; O=12; E=5.74; R=2.09 | 0.0133 | 0.033 |
| MIR-136 | AATGGAG | [5](file:///C:\Users\TEMP\Rar$EXa0.573\miRNA1446574303\final_mirna_geneset_file_1446574303.html#hsa_AATGGAG,MIR-136) | 2186 345557 29123 5892 23196 | C=75; O=5; E=1.45; R=3.46 | 0.0149 | 0.0364 |
| MIR-183 | GTGCCAT | [8](file:///C:\Users\TEMP\Rar$EXa0.573\miRNA1446574303\final_mirna_geneset_file_1446574303.html#hsa_GTGCCAT,MIR-183) | 55740 4154 64388 7091 7430 27436 8473 6749 | C=169; O=8; E=3.26; R=2.46 | 0.017 | 0.041 |
| MIR-365 | GGGCATT | [6](file:///C:\Users\TEMP\Rar$EXa0.573\miRNA1446574303\final_mirna_geneset_file_1446574303.html#hsa_GGGCATT,MIR-365) | 26057 29123 6645 8861 10000 58516 | C=107; O=6; E=2.06; R=2.91 | 0.0175 | 0.0417 |
| MIR-196A,MIR-196B | ACTACCT | [7](file:///C:\Users\TEMP\Rar$EXa0.573\miRNA1446574303\final_mirna_geneset_file_1446574303.html#hsa_ACTACCT,MIR-196A,MIR-196B) | 10643 96459 10771 6433 5903 6599 64062 | C=141; O=7; E=2.72; R=2.58 | 0.0196 | 0.0461 |
| MIR-142-5P | ACTTTAT | [11](file:///C:\Users\TEMP\Rar$EXa0.573\miRNA1446574303\final_mirna_geneset_file_1446574303.html#hsa_ACTTTAT,MIR-142-5P) | 55082 51773 10725 10643 55137 7091 80821 206358 22862 25778 6651 | C=284; O=11; E=5.47; R=2.01 | 0.0224 | 0.0488 |
| MIR-34B | ACTGCCT | [9](file:///C:\Users\TEMP\Rar$EXa0.573\miRNA1446574303\final_mirna_geneset_file_1446574303.html#hsa_ACTGCCT,MIR-34B) | 23077 23321 10725 5514 392 6433 59338 6659 7029 | C=211; O=9; E=4.07; R=2.21 | 0.0216 | 0.0488 |
| MIR-198 | TCTGGAC | [5](file:///C:\Users\TEMP\Rar$EXa0.573\miRNA1446574303\final_mirna_geneset_file_1446574303.html#hsa_TCTGGAC,MIR-198) | 23389 10618 23008 55137 83439 | C=82; O=5; E=1.58; R=3.16 | 0.0211 | 0.0488 |
| MIR-202 | ATACCTC | [8](file:///C:\Users\TEMP\Rar$EXa0.573\miRNA1446574303\final_mirna_geneset_file_1446574303.html#hsa_ATACCTC,MIR-202) | 55284 80155 23389 2589 800 9877 11214 867 | C=178; O=8; E=3.43; R=2.33 | 0.0224 | 0.0488 |
| MIR-451 | AACGGTT | [2](file:///C:\Users\TEMP\Rar$EXa0.573\miRNA1446574303\final_mirna_geneset_file_1446574303.html#hsa_AACGGTT,MIR-451) | 8473 51719 | C=12; O=2; E=0.23; R=8.65 | 0.0215 | 0.0488 |
| MIR-21 | ATAAGCT | [6](file:///C:\Users\TEMP\Rar$EXa0.573\miRNA1446574303\final_mirna_geneset_file_1446574303.html#hsa_ATAAGCT,MIR-21) | 55082 4154 91452 6433 59338 57542 | C=113; O=6; E=2.18; R=2.75 | 0.0223 | 0.0488 |
| MIR-378 | GTCAGGA | [4](file:///C:\Users\TEMP\Rar$EXa0.573\miRNA1446574303\final_mirna_geneset_file_1446574303.html#hsa_GTCAGGA,MIR-378) | 55284 55704 10645 8473 | C=56; O=4; E=1.08; R=3.71 | 0.0227 | 0.0489 |
|  |  |  |  |  |  |  |
| *Number of predicted target genes; ^#^*p-value* from hypergeometric test; ^&^ Adjusted *p-value* by Benjamini & Hochberg correction; C: number of reference genes in the category; O: number of genes in the gene set and in the category; E: expected number in the category; R: Ratio of enrichment. In bold miRNAs selected for further analysis. | | | | | | |
