# Supplementary material for: MiR-9, miR-153 and miR-124 are down-regulated by acute exposure to cocaine in a dopaminergic cell model and may contribute to cocaine dependence
Source: Transl Psychiatry. 2018 Aug 30;8:173. doi: 10.1038/s41398-018-0224-5 (PMC6117282; doi:10.1038/s41398-018-0224-5)
Supplement: Supplementary file 2 — Supplementary Table 2 [file 41398_2018_224_MOESM2_ESM.docx]

| **Supplementary table 2**. Primer sequences used in qRT-PCR assays. | | |
| --- | --- | --- |
|  |  |  |
| **miRNA** | **miRNA sequence** | **Primer sequence (5’🡪3’)** |
| hsa-124-3p | uaaggcacgcggugaaugcc | taaggcacgcggtgaatgcc |
| hsa-124-5p | cguguucacagcggaccuugau | cgtgttcacagcggaccttgat |
| hsa-miR-9-5p | ucuuugguuaucuagcuguauga | tctttggttatctagctgtatg |
| hsa-miR-9-3p | auaaagcuagauaaccgaaagu | ataaagctagataaccgaaagt |
| hsa-miR-369-3p | aauaauacaugguugaucuuu | gaataatacatggttgatcttt |
| hsa-miR-137 | uuauugcuuaagaauacgcguag | ttattgcttaagaatacgcgta |
| hsa-miR-186-5p | caaagaauucuccuuuugggcu | caaagaattctccttttgggc |
| hsa-miR-101-3p | uacaguacugugauaacugaa | tacagtactgtgataactgaa |
| hsa-miR-153-3p | uugcauagucacaaaagugauc | ttgcatagtcacaaaagtgatc |
| hsa-miR-105-5p | ucaaaugcucagacuccuguggu | tcaaatgctcagactcctgtg |
